# Optical coherence tomography angiography (OCTA) as a new diagnostic tool in uveitis

**DOI:** 10.1186/s12348-019-0176-9

**Published:** 2019-05-28

**Authors:** Vita L. S. Dingerkus, Marion R. Munk, Max P. Brinkmann, Florentina J. Freiberg, Florian M. A. Heussen, Stephan Kinzl, Sandra Lortz, Selim Orgül, Matthias Becker

**Affiliations:** 1 Department of Ophthalmology, City Hospital Triemli, Birmensdorferstrasse 497, CH-8063 Zürich, Switzerland; 2 Department of Ophthalmology, University Clinic Bern, Bern, Switzerland; 3 Department of Ophthalmology, University of Heidelberg, Heidelberg, Germany

**Keywords:** Optical coherence tomography angiography, Fluorescein angiography, Indocyanine angiography, Uveitis, Diagnostic tools

## Abstract

**Background:**

The broad spectrum of uveitis disorders requires a multimodal imaging approach in the daily practice of an ophthalmologist. As inflammatory conditions, they have in common an alteration in leukocyte migration. In this context, optical coherence tomography angiography (OCTA) might be of great value for diagnosing or following up patients with these disorders. To date, OCTA has rather been used as an additional tool besides the well-established diagnostic imaging tools, but its complementary diagnostic features become increasingly relevant, to follow disease activity and treatment response and for the understanding of pathomechanisms of various uveitis types.

This review summarizes the possible applications of OCTA and its advantages and disadvantages as opposed to dye-based angiographies in uveitic diseases.

**Main body:**

Hitherto gold standards in the diagnostic workup of posterior or intermediate uveitis have been angiography on a dye-based method, which is fluorescein or indocyanine green. It gives information about the status of the blood-retinal barrier and the retinal and choroidal vasculature by visualizing diffuse leakage as a state of inflammation or complications as an ischemia or choroidal neovascularization. As noninvasive methods, fundus autofluorescence depicts the status of metabolic activity of the retinal pigment epithelium and OCT or enhanced depth imaging OCT, respectively, as a depth-resolving imaging method can supply additional information.

OCTA as a non-invasive, depth-resolution imaging tool of retinal and choroidal vessels adds detailed qualitative and quantitative information of the status of retinal and choroidal vessels and bridges the gap between the mentioned conventional diagnostic tools used in uveitis.

It is important, though, to be aware of its limitations, such as its susceptibility to motion artifacts, limited comparability among different devices, and restricted contribution of information regarding the grade of disease activity.

**Conclusion:**

OCTA as a non-invasive, depth-resolution imaging tool can give qualitative and quantitative information about the status of retinal and choroidal vessels, but also has certain limitations. Employing OCTA as a complementary rather than exclusive tool, it can give important additional information about the macro- and microvasculature under inflammatory circumstances. Thereby, it also contributes to the understanding of the pathophysiology of various uveitis entities.

## Background

Optical coherence tomography angiography (OCTA) is a new diagnostic tool which was developed as an extended technique of already existing imaging module. Optical coherence tomography (OCT) produces a depth-resolved evaluation of the reflectance data from tissues. OCT thereby allows a three-dimensional image of the retina, which is also the case for OCTA [1, 2]. OCTA employs speckle or phase variance or amplitude decorrelation for detection of motion contrast and imaging of blood flow of different layers of the retina and choroid. Stationary tissue, in contrast, does not lead to a signal in OCTA, whereas changing structures, as is the case in blood flow, can be detected via repeated images over the same focus of interest [3]. In contrast to dye-based angiographies, OCTA is non-invasive, non-hazardous, and easier and faster to apply. This makes OCTA very interesting, especially in the field of retinology and uveitis.

In uveitis, various vascular abnormalities can be seen due to different underlying pathomechanisms, leading to ischemia, neovascularization, or retinal and/or choroidal vasculitis. Up to now, most important diagnostic tools for vascular pathologies are fluorescein angiography (FA) and indocyanine green angiography (ICGA), both requiring an infusion of dye, thereby being invasive and holding the risk of side effects [4]. In various conditions, such as known allergy against the substances, renal impairment, or pregnancy, the use of dye should be avoided or only used with caution. Furthermore, these techniques are limited when it comes to the warrant of further evaluation of different vascular layers than only distinction of retinal and choroidal vasculature [5, 6]. Moreover, in FA and ICGA images, the visual accessibility of vessels can be limited and hampered by dye leakage. Non-invasive fundus autofluorescence images (FAF), OCT, and enhanced depth imaging-OCT (EDI-OCT) can supply additional information about the structures of concern, but do not show details of the vascular structure or blood flow. OCTA, in contrast, can visualize the different layers more independently from surrounding leakage of fluorescein, than FA [7, 8]. However, the quality of OCTA images can also be reduced due to an overlying obscuration like in FA, for example, in cases of vasculitis, predominantly by vitritis or bleedings.

Finally, as the central vision might be affected, the assessment of OCTA images can become quite difficult [9].

Nevertheless, OCTA might be an additional tool in a multimodal imaging approach to provide a better understanding of various uveitis types; however, because of its complexity and susceptibility to artifacts, it requires careful scrutiny during interpretation [10].

This review aims to give an overview on new knowledge gained so far about the applicability and limitations of OCTA in different forms of uveitis. Advantages or disadvantages, when it comes to comparison of OCTA and dye-based angiographies, will be highlighted. It emphasizes the use of OCTA in posterior uveitis and white dot syndromes, as it is in these entities, that most studies and reports have been published. However, it must be mentioned that a major proportion of publications on OCTA and uveitis is based on case reports or case series.

## About fluorescein angiography

Fluorescein sodium is a dye containing fluorescein molecules of 354 Da which is injected intravenously to obtain an image of the vascular status of the retina. Under inflammatory conditions, the unbound part (ca. 20%) can leak out of retinal vessels, even the smallest ones, thereby making it the first-line-investigation for retinal vasculitis, capillary leakage, and edema, either diffuse or focal, or for identification of choroidal neovascularization [11–15].

Nowadays, the gold standard for diagnosing macular edema (ME) is OCT, as it is, in contrast to fluorescein angiography (FA), noninvasive, faster, and easier applicable. However, it should be considered to be complementary to FA, as the latter may show leakage when retinal thickening is not verifiable [16]. Thus, FA remains the most sensitive modality to detect retinal vessel inflammation and allows monitoring the process of the disease or response to therapy. In particular, widefield FA is of interest in uveitis because it can detect peripheral vascular leakage, ischemic conditions, or nonperfusion and is highly specific and sensitive for clinical disease activity [17, 18]. Considering uveitis, FA has become an essential diagnostic tool for intermediate and posterior uveitis. It is important for anterior uveitis in case of co-existing macular edema. However, FA has not been established as an essential imaging tool for anterior vascular affection although it may detect subtle peripheral and optic nerve head leakage in active anterior uveitis as well [19].

In addition to the need of an invasive injection of dye to the patient, there are risks of side effects. Besides the risk of local complications such as tissue necrosis, the risk of general side effects, reaching from moderate nausea to severe allergic reaction, needs to be taken into account [4]. One major disadvantage in terms of image resolution is its limitation in unveiling the morphological details of vessels and their anatomical layers, especially when there is leakage [20, 21]. Moreover, deeper choroidal vessels cannot be evaluated properly, as the choriocapillaris is fenestrated and naturally leaks dye, and, thus, its own vascular details and the larger choroidal vessels are obscured [20].

## About indocyanine angiography

While fluorescein is a rather small molecule and mainly unbound, indocyanine green (ICG) is mostly (98%) bound to plasma proteins. Therefore, it is mainly used to evaluate the choroidal vasculature, as it remains in the vessels, unlike fluorescein, whose wastage from the intravasal space makes appraisal of the choroidal vascular morphology impossible [20].

ICGA thus plays an important role in diagnosing diseases including damage of the retinal pigment epithelium (RPE) and serous detachment [20, 22], and has been involved in diagnostic criteria of inflammatory diseases, mainly those affecting or originating from the choroid, such as the “white dot syndromes” (such as multiple evanescent white dot syndrome (MEWDS), acute posterior multifocal placoid pigmentepitheliopathy (APMPPE), multifocal choroiditis and panuveitis (MCP) and punctate inner choriodopathy (PIC), serpiginous choroiditis, and birdshot chorioretinopathy (BSR)), or granulomatous inflammations such as sarcoidosis and tuberculosis as well as other infectious diseases such as syphilis or toxoplasmosis [23].

Considering certain ICGA patterns, one can differentiate between choriocapillaritis (seen in white dot syndromes) and stromal choroiditis, mostly granulomatous [24].

In choriocapillaritis, hypocyanescence on ICGA can either be a sign of hypoperfusion of the choroid, appearing in a patchy or geographic pattern, or an impairment of the filling of the choroid with the dye due to space-occupying lesions [24]. In the convalescent phase, this hypocyanescence may diminish or remain in case of chorioretinal atrophy (corresponding to window defect on FA) [24]. Progressing lesions such as in serpiginous choroiditis may show diffuse hypercyanescence at the edges of lesions [24].

Stromal choroiditis can be full- and partial thickness inflammation (mostly granulomata). It can either reflect a choroid targeting disease or be part of a systemic condition, in which case the hypocyanescent spots are distributed less regularly [23]. For example, in the case of birdshot chorioretinopathy, Vogt-Koyanagi-Harada disease and sympathetic ophthalmia hypocyanescent dots are evenly distributed, whereas in sarcoidosis, those spots appear in a more irregular pattern; both remaining hypocyanescent till the late phase, unless there is only partial thickness of the choroid affected, in which case it would become isocyanescent, because the choriocapillaris is not affected [23]. Late hypercyanescent spots surrounding those dark spots are a sign of surrounding larger choroidal inflamed vessels [23].

Furthermore, hypocyanescence may be a sign of diseased RPE, as a result of reduced indocyanine green uptake by the retinal pigment epithelium, which is important for co-interpretation of OCTA and ICGA, especially in diseases, where the question rises, whether the RPE or the choroid is the primary focus of inflammation, as described below [21, 25].

Just as in FA, interpretation and performance in ICGA are limited to its two-dimensionality, the need of injection, and the risk of side effects [26].

## About optical coherence tomography angiography

After OCT had fundamentally changed the diagnostic approach in ophthalmology over the last two decades, optical coherence tomography angiography (OCTA) has been introduced into the field of ophthalmology only a few years ago. The technique of OCTA is based on the comparison of repeatedly assessed OCT scans of the same region, imaging the change between images, which is, in case of OCTA, the movement of blood in the vasculature of the eye. This is important to consider, because what OCTA depicts as a vessel is actually moving blood within the inner, vascular lumen. Further, OCTA will depict all moving particles, thus also suspended scattering particles in motion (SSPiM), regularly seen in exudative maculopathies [27].

OCTA provides—in contrast to dye-based imaging tools—depth resolution images visualizing the deep and superficial vessels as well as the middle capillary plexus and choriocapillaris [5, 6, 28]. Swept source OCTA will be also able to exhibit deeper choroidal vessels. It provides a high segmentation ability (1- to 10-μm resolution), which is based on the concept known as inferometry using an optical fiber-based system [3, 28]. Hence, OCTA gives us the possibility to image the different retinal and choroidal vascular layers in multiple en face images, as opposed to single en face view in FA and ICGA.

It is non-invasive and, for ophthalmological use, mainly based on three OCTA techniques: speckle variance, amplitude decorrelation, or phase variance [28]. Speckle variance quantifies the changes in speckle pattern to visualize motion, that is, a higher variance of amplitude changes with flow than in a non-moving sample [28].

Similarly, in amplitude decorrelation, the amplitude of a signal is used for correlation as a metric to detect flow. In case of flow, the decorrelation of the amplitude calculated between the B-scans is higher than in a non-moving sample [28].

Contrarily, phase variance does not include amplitude variations, but only changes in phase. The variance is higher in the presence of flow than in static areas [28].

Like any other imaging device, OCTA has limitations in its applicability and validity. One major issue is artifacts.

A false positive flow can be due to eye and head movement, which can lead to artifacts that make the picture “noisy,” and should be avoided as much as possible by using eye tracking systems. However, the corrected pictures may have the appearance of a patchwork and show vascular doubling [10]. Furthermore, low flow systems (choroid or vitreous) can cause noise [29]. The artifact of noise can tentatively be prevented by introducing signal threshold levels, yet this counteraction itself may lead to attenuation artifact-induced interpretation errors [29]. Reduction of background noise is manufacturer specific and the procedure is not always transparent for the user, and can lead to signal loss as for the case of smaller vessels, whose already diminished signal might then be further attenuated [29].

False-negative flow due to low decorrelation happens when the blood flow is too slow or too fast [10]. It can also be caused by projection artifacts beneath the perfused vasculature targeted in the investigation, because the reflected light passing through overlying vessels varies over time and changes the reflection of underlying structures [6]. Thus, at the level of RPE or choroid, one would still see retinal vessels [6]. Additionally, in case of media or other pathological opacities, such as aggregations in the vitreous, placoid chorioretinitis, or chorioretinal granulomata, a shadowing artifact can occur [10]. Dry eyes may be a problem, leading to media opacity, but the quality of the image can be improved by optimal lubrification [29]. Another issue may be cataract and anterior segment inflammation, which may lead to false low quantitative measurements despite good image quality [30]. But, of course, these media opacities may also impair the quality of other diagnostic imaging.

Further, due to a fixed interscan time, only blood flow at a certain speed can be captured and blood flux but not the speed of blood flow may be captured with commercially available devices [31]. Another limitation includes the limited comparability among different devices due to different methods used to generate flow motion but also due to different segmentation-approached algorithms and artifact removal algorithms [32, 33].

Finally, as compared to dye-based angiography which can obtain ultra-widefield images, OCTA is limited when it comes to the assessment of peripheral findings with a maximum of 15 × 9 or 12 × 12 mm in swept-source OCTA (SS-OCTA), as further increasing image size would mean a tradeoff in image quality [34]. To date, widefield images can only be obtained by montaging OCTA images of a smaller field [34]. Montaging software is still in the progress of development.

## About the implementation of optical coherence tomography angiography as a diagnostic tool in uveitis

Vessels of different structures in the eye are involved in or are the origin of different types of uveitis. Until now, the gold standard for diagnosing and managing uveitis has been FA and ICGA, as for assessing disease activity and imaging the retinal or choroidal vasculature. OCT is used to capture ME, and autofluorescence imaging to detect involvement of the RPE and outer retina.

As mentioned above, FA and ICGA have several limitations for their validity, especially when it comes to the quest of understanding the three-dimensionality and the pathohistological correlates.

OCTA has not yet been established, but gets more and more involved as another complementary imaging tool to fill some of the gaps that are left by the conventional angiographies.

### About optical coherence tomography angiography in anterior uveitis

OCTA devices, as an additional algorithm of the OCT, can obtain information about the posterior, but also the anterior segment; yet, studies on the application of OCTA in eyes with anterior uveitis are still quite rare [19, 35–38]. Similar to FA, OCTA is incapable of visualizing the ciliary body, which is also affected in anterior uveitis, but it can visualize iris hyperemia and iris neovascularization, as a possible complication in anterior uveitis, and would, theoretically, allow a quantitative and objective follow-up for patient at risk for neovascularization of the iris [39]. Limitations are set by pupil diameters, iris color diversity, and optical distortion caused by anterior tissue refraction of the OCT beam, especially the cornea [19, 39].

In a cross-sectional observational clinical study, anterior segment OCTA was evaluated in 20 normal irides to analyze anterior segment and iris vasculature [40]. In this study, the usability of OCTA was found to be beneficial in ruling out ischemic conditions and in detecting iris and ciliary body tumors, as well as in traumatic and surgical conditions of the anterior segment, since eyes with a history of uveitis were excluded [40].

Compared to FA, iris pigmentation is still a problem in OCTA, because it blocks imaging of the iris vasculature, but OCTA provides better quality images in darker irides and more pigmented areas of the iris compared to FA [40].

One study quantified retinal capillary density in uveitic eyes and found decreased parafoveal capillary density not only in intermediate and posterior uveitis, but also in eyes with anterior uveitis irrespective of presence or absence of a ME [36].

### About optical coherence tomography angiography in intermediate uveitis

Intermediate uveitis (IU), defined by an intraocular inflammation mainly apparent in the vitreous and at the peripheral retina, cannot directly be pictured by OCTA, as the main affected structures, the pars plana or the ciliary body, are out of its reach.

In a case-control study (29 eyes with IU versus 30 healthy eyes), reduced vascular density and complexity in superficial as well as deep retinal layers and altered choriocapillaris perfusion were present in patients with IU, meaning that macular microvasculature—regardless if ME was present—was altered [41].

In a prospective cross-sectional study, 156 eyes, including 120 with IU and 36 with concomitant vasculitis, were evaluated using widefield swept-source SS-OCTA [42]. OCTA capillary non-perfusion and reduced perfusion were more frequently observed in the choroid, choriocapillaris, and deep capillary plexus (DCP) than in the superficial capillary plexus (SCP), and no association was observed between peripheral capillary leakage on FA and non-perfusion or reduced peripheral perfusion on widefield OCTA [42].

At the same study site, another prospective, cross-sectional study using SS-OCTA was performed to evaluate vascular changes in patients with intermediate uveitis with or without retinal vasculitis evaluating OCTA scans (widefield montage and central 3 × 3 mm slabs) of 93 eyes of 58 patients with IU and 33 healthy age-matched controls [43]. Although wide field images could give important information about peripheral vascular changes, their lower resolution limits the detectability of flow alterations compared to the high resolution of the central scans. Microvascular changes were in most IU cases found at the level of the SCP and DCP, the choriocapillaris and the choroid and change of central vascular density found in the central scans did not correlate to alterations found on wide field OCTA [43]. It was also shown that rather the presence of an epiretinal membrane and a ME than the disease entity had an effect on the FAZ size [43].

In summary, intermediate uveitis, with or without associated vasculitis and in the presence or absence of ME and ERM, leads to an alteration and decrease of the retinal parafoveal capillary density and to an alteration of the FAZ. Capillary changes may be also found beyond the posterior pole on wide field OCTA. Interestingly, not only the retinal vasculature seems to be altered but also the choriocapillaris and the choroid, which definitely warrants further evaluation. Data on active eyes are sparse and are needed as well to figure out whether indirect signs of disease activity can be visualized using OCTA.

### About optical coherence tomography angiography in posterior uveitis

The definition of posterior uveitis includes focal, multifocal, or diffuse choroiditis, chorioretinitis, retinochoroiditis, retinitis, and neuroretinitis [44]. They can be infectious or noninfectious, be part of a systemic disease, or limited to the ocular appearance as an ocular syndrome.

These findings are, as mentioned earlier, commonly diagnosed with the help of dye-based angiographies, in addition to funduscopy, and, in some uveitis types, autofluorescence imaging.

Vasculitis, also a common sign of various forms of posterior uveitis, is defined as retinal vascular nonperfusion, retinal hemorrhages, vascular sheathing, overlying vitritis, and leakage on fluorescein angiography [44]. As for affection of the arterial system, vasculitis can be caused by a systemic vasculitis, for example, polyarteritis nodosa, systemic lupus erythematosus, or granulomatosis with polyangiitis, formerly Wegener’s granulomatosis, but also by Behçet’s disease. If the venular branch of the vascular arcades predominantly shows signs of vasculitis (like sheathing or inflammatory occlusion), the condition is rather associated with sarcoidosis, multiple sclerosis, or tuberculosis, but can also be seen in primary intermediate uveitis or in birdshot [8].

Though there may be various restrictions in OCTA image quality in vasculitis such as vitritis or bleeding, OCTA can be a useful or even superior imaging tool. In some vasculitis cases, neovascularization obscured by retinal hemorrhage, early peripapillary neovascular proliferation, or telangiectasia were better detectable than in FA, as shown in an observational case series of 19 eyes with various uveitis types causing vasculitis [9].

Most of all, OCTA seems to be important in revealing capillary non-perfusion areas in retinal vasculitis. As concluded in a prospective, comparative, cross-sectional study with 44 eyes and a smaller retrospective case series, OCTA unveils microvascular loss of flow in areas affected by vasculitis as diagnosed via FA, which were undetectable with funduscopy, as funduscopic signs of ischemia, such as cotton wool spots or faint retinal whitening, are not present [7, 8]. In a case of occlusive vasculitis in a West Nile virus infection, OCTA was even estimated to be superior to FA, in detecting ischemic retinal capillary changes [45].

In Behçet’s retinal vasculitis, OCTA revealed that the DCP is more affected than the superficial layer, as shown in an analytic cross-sectional study including 37 eyes [46]. This was confirmed by a study group, which tested an algorithm for OCTA by mapping the perifoveal region with two concentric circles, using diameters of 1 and 3 mm; furthermore, these circles were divided into superior, inferior, temporal, and nasal regions and categorized into DCP and SCP, the latter being additionally divided into small and capillary vessels [47]. They revealed that hypoperfusion, mainly in the inferior macular region, is most affected, with the DCP being more affected than the SCP in patients with Behçet’s disease [47]. The finding of low capillary vessel density with DCP being more affected was affirmed by a very recent study evaluating 32 eyes of patients compared to 30 healthy eyes [48].

This is of significant importance, as in patients with Behçet’s disease, the foveal avascular zone cannot conclusively be assessed by FA because of dye leakage, due to vasculitis. Even in the eyes of patients that are categorized as eyes without ocular involvement by conventional imaging techniques, OCTA can reveal microvascular changes, as shown by a conducted investigation of 20 eyes [49]. Particularly, all eyes showed parafoveal capillary telangiectasia in the DCP, and a majority of cases showed capillary retinal hypoperfusion on OCTA [49]. Thus, OCTA seems to be a more reliable technique for monitoring these patients than FA, which is important in detecting early risk of central loss of vision [7, 9, 46]. However, when scrutinizing possible artifacts, one has to consider the restriction in interpretation regarding false-negative flow: in this case, a too slow flow could also be the reason for a false-negative darkening in the OCTA images, possibly explaining the missing funduscopic signs of ischemia [7, 10].

Another sign of posterior uveitis may be granulomata of the retina and choroid, which can be part of a systemic granulomatous disease, such as tuberculosis, sarcoidosis, Lyme disease, or syphilis. Granulomata, considered from a histopathological point of view, usually consist of macrophages and epithelioid cells encircled by lymphocytes forming nodules, caseating, as in the case of tuberculosis, or non-caseating, as, for example, in sarcoidosis. From the clinical point of view, so-called granulomata in the anterior chamber do not necessarily meet these histological criteria. Small granulomata of the retina are not visible in OCTA, but full-thickness granulomata can be visible in the form of hyporeflectivity, corresponding to reduced flow [19].

Looking at sarcoidosis more specifically, besides the OCTA results for granulomata, OCTA findings of the SCP and DCP, as well as the choriocapillaris, seem to be similar to those described in Behçet’s disease: a case-control, cross-sectional observational study with 19 eyes of 12 patients and 19 healthy control eyes showed a higher sensitivity of OCTA than FA in assessing macular microvascular abnormalities [50], with the DCP appearing more affected by hypo-perfusion/non-perfusion than the SCP, but with the SCP being more affected by the presence of capillary abnormalities. Visual acuity showed a positive correlation with the capillary vessel density at the level of the SCP, which might explain the involvement of the SCP at an advanced stage of the disease [50]. The choriocapillaris also showed flow void areas on OCTA in 42.1% of the sarcoidosis eyes, likely explained by active granulomata, chronic tissue damage secondary to previously active granulomata (mechanically), or the presence of focal choroidal arteriolitis [50].

Deep choroidal granulomata, such as in Vogt-Koyanagi-Harada disease (VKH), visible on ICGA and EDI-OCT, cannot be assessed by conventional OCTA. However, hypoperfusion in the overlying areas of the choriocapillaris can be detected by OCTA, as shown in a prospective study with ten affected eyes [51]. In turn, combined with ICGA and EDI-OCT, the technique of OCTA was considered to be a convenient imaging tool not only to diagnose, but also to follow-up cases of acute VKH, follow treatment response, and depict recurrences and reduce the use of ICGA [51].

### OCTA in secondary complications of uveitis

**Fig. 1:**
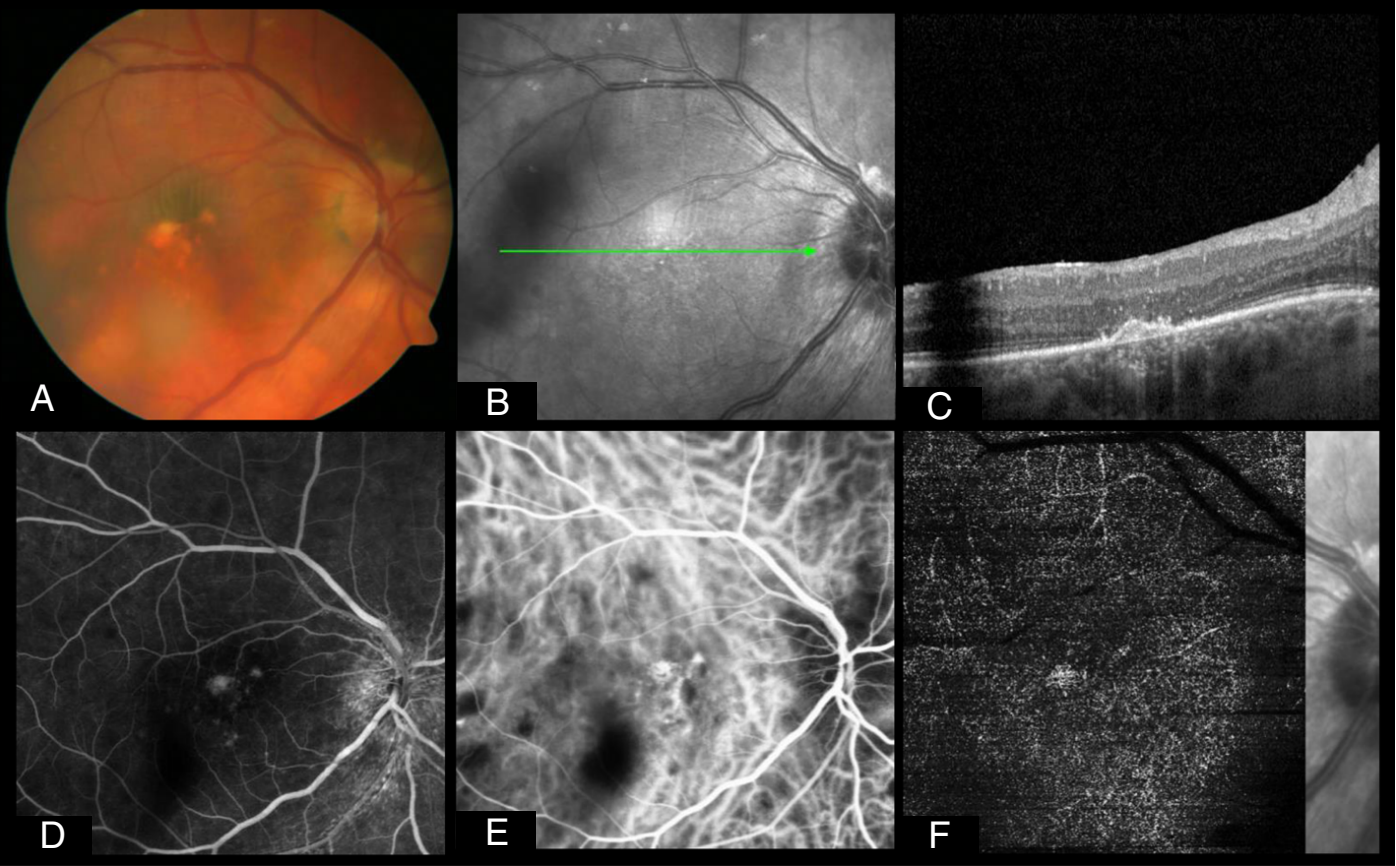
This patient diagnosed with birdshot retinopathy had been treated with intravitreal steroids abroad due to uveitic macular edema prior to final diagnosis. At first presentation, vision was already decreased to 20/100 on the right eye and 20/200 on the left eye, respectively. On both eyes, there was central atrophy of outer retinal layers besides typical yellow-white lesions on the fundus (**a**) and furthermore persisting macular edema on the left eye (not shown). These pictures reveal the presence of a choroidal neovascularization (CNV) of the right eye, although there was no intraretinal fluid present on the optical coherence tomography (OCT) scan around a suspicious lesion (**b** and **c**). Fluorescence angiography peak phase shows light hyperfluorescence of this lesion besides smaller hyperfluorescent spots corresponding to small pigment epithelial detachments (**d**). On indocyanine green angiography, the birdshot lesions are typically hypocyanescent, but here, central hypercyanscence can be noted (**e**). OCT angiography confirms a quiescent subretinal (type 1) CNV (**f**)

**Fig. 2:**
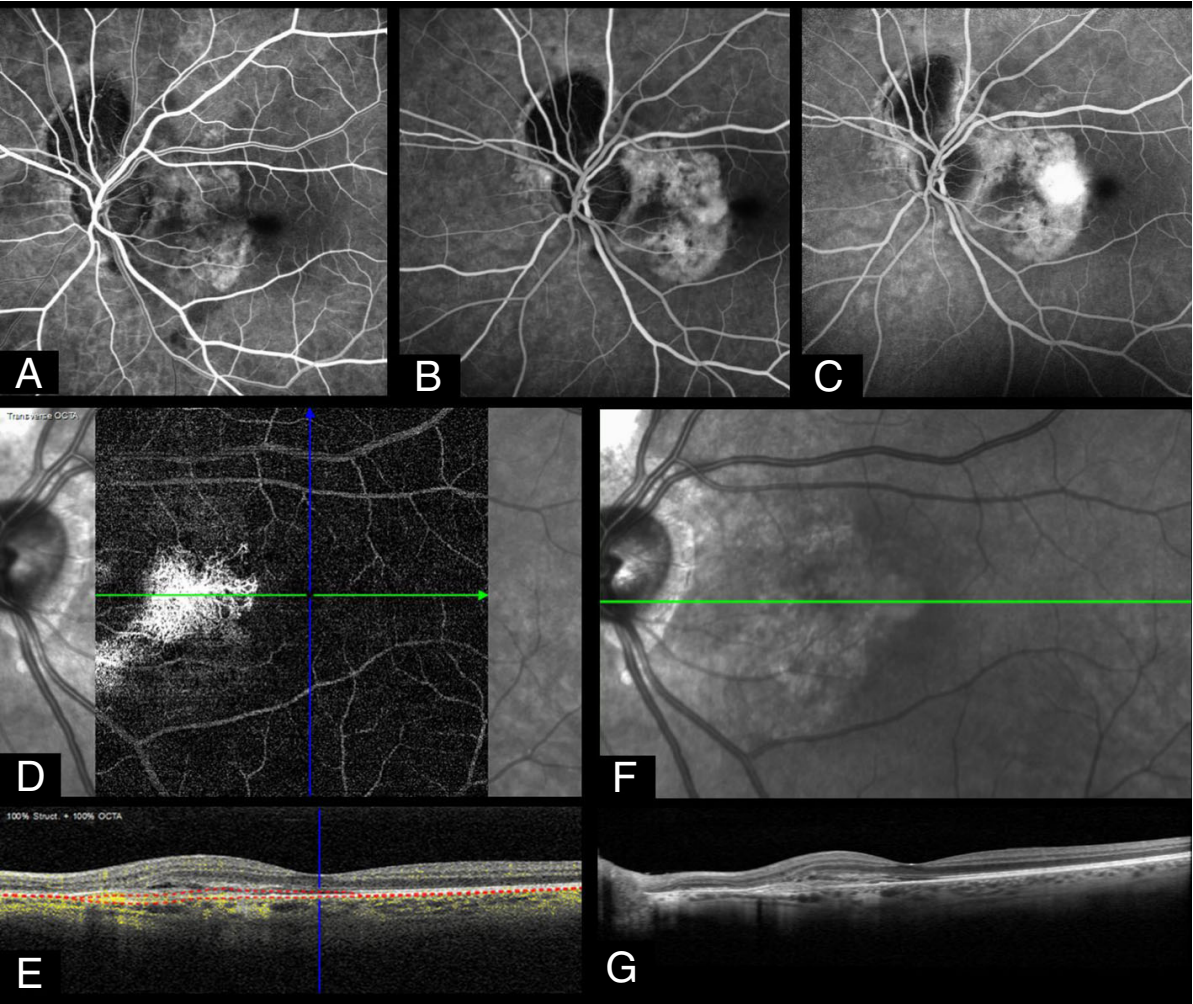
This 49-year-old lady presented with a 6-week history of reduced vision in the left eye. Visual acuity at presentation was 6/12 in the affected eye, which also showed a chorioretinal scar from a past toxoplasmosis-related chorioretinitis. Fluoresceine angiography revealed a parafoveal spot of active leakage from early- (**a**) through mid- (**b**) to late-phase (**c**). Optical coherence tomography angiography (OCTA) confirmed the presence of a vascular network (**d**) between the retinal pigment epithelium and Bruch’s membrane (**e**). Structurally, this corresponded to pigment changes on infra-red imaging (**f**) and subretinal fluid next to a fibrovascular pigment epithelial detachment on OCT (**g**)

One of the most frequent complications in posterior or panuveitis is the development of choroidal neovascularization (CNV), disrupting Bruch’s membrane and resulting in central vision loss, if untreated. The underlying disease can be of infectious or noninfectious origin, usually associated with scar formation, lesions, or choroidal granulomata [15]. Based on classification in age-related macula disease, CNV can be located subretinally (type 1), in the outer retina (type 2), or both (mixed type). Inflammatory CNVs are mostly detected as type 2 CNV; however, a minority of patients may also exhibit a type 1 component [15, 52, 53]. One problem in diagnosing inflammatory CNV with FA is the possible similar appearance of CNV (early iso- or hyperfluorescence and late hyperfluorescence due to leakage) and inflammatory lesions (early iso- or hypofluorescence and late hyperfluorescence due to leakage in active or staining in inactive lesions) [15]. To differentiate these conditions, ICGA and OCT are commonly used for additional diagnosing and follow-up of inflammatory CNVs. However, as a secondary, inflammatory CNV does not necessarily present with a sub- or intraretinal fluid on OCT and may just show an increase in subretinal hyperreflectivity on OCT, further assessment tools are warranted. OCTA was identified as a very helpful tool to distinguish inflammatory lesions from CNV [54–56] (Figs. 1 and 2). Moreover, in several case series, OCTA was helpful in detecting CNV, where FA did not provide any hints, especially when considering type 1 CNV [53, 57]. Significantly more inflammatory CNVs are detected using OCTA than FA; up to 83% MCP/PIC lesions present flow changes consistent with a CNV [55–57]. A useful collection of the most important references regarding this topic can be found in a review written by Pichi et al. [19].

Another common complication in IU is ME, which, up to now, was a favored focus of previous OCTA studies.

A prospective observational OCTA study including 74 eyes separated into five groups (posterior uveitis with or without ME, anterior uveitis with or without ME, and healthy controls) showed that, when compared to healthy controls, eyes with non-infectious posterior uveitis with or without ME had a significantly larger deep foveolar avascular zone, and eyes with anterior uveitis and ME had a significantly larger deep and superficial foveolar avascular zone [58]. A possible explanation for these findings might be a peripheral displacement of retinal capillaries as a result of the formation of cystoid spaces or cysts, preferentially leading to non-perfusion areas [58].

Uveitic ME was associated with significant changes in the deep retinal layer, regardless of projection artifacts, correlating well with the location of intraretinal cystoid spaces in the inner retina (generally inner nuclear and plexiform layers), as shown by a cross-sectional, observational case series of eyes with anterior, posterior, or panuveitis and healthy controls (but no cases of intermediate uveitis) [36]. This is thought to represent an actual change in the capillary density owing to ME, but a physical displacement of retinal capillaries by the ME remains a plausible interpretation [36]. It was summarized that qualitative and quantitative changes in the parafoveal capillary density and morphology of subjects with uveitis can reliably be detected using OCTA [36].

### About optical coherence tomography angiography in white dot syndromes

There are multiple publications on OCTA in the clinical spectrum of so-called white dot syndromes, a heterogenous group of various forms of posterior or panuveitis having light fundus lesions as at least one common clinical distinguishing sign. The group of diseases that constitute the white dot syndromes include acute posterior multifocal placoid pigment epitheliopathy (APMPPE), serpiginous choroiditis, multiple evanescent white dot syndrome (MEWDS), multifocal choroiditis and panuveitis (MCP), punctate inner choroidopathy (PIC), diffuse subretinal fibrosis (DSF), presumed ocular histoplasmosis syndrome (POHS), and birdshot retinochoroidopathy (BSR). Also, acute macular neuroretinopathy (AMN) and acute zonal occult outer retinopathy (AZOOR) belong to the category of white spot syndromes.

To date, quite a number of reports on OCTA in white dot syndromes have been published (except for DSF), but, given the low incidence of these diseases, most are case series or case reports (see Table 1).

**Table 1:** Summary of publications on the implementation of OCTA in white dot syndromes

Disease	Publication	Number of eyes/patients	Study type	Main results and points of discussion	OCTA device
MCP and PIC	Zahid S, Chen Kc, Jung Jj, Balaratnasingam C, Ghadiali Q, Sorenson J, Et Al. Optical coherence tomography angiography Of chorioretinal lesions due to idiopathic multifocal choroiditis. Retina. 2017;37(8):1451–63.	18 eyes of 14 patients with MCP	Descriptive, retrospective study	OCTA flow signals consistent with neovascularization were identifiable in 83% of eyes including 0% of subretinal pigment epithelium, 91% of subretinal, and 100% of mixed lesions OCTA also identified CNV in cases where FA was inconclusive for the presence of CNV No change in quantitative measurements of greatest linear diameter, area, and largest vessel diameter of the neovascular complex following anti-VEGF therapy was observed	Optovue Avanti-RTVue-XR (Optovue, Fremont, California, USA) or Cirrus Angioplex OMAGC (Carl Zeiss Meditec Inc)
	Levison Al, Baynes Km, Lowder Cy, Kaiser Pk, Srivastava Sk. Choroidal neovascularisation on optical coherence tomography angiography in punctate inner choroidopathy and multifocal choroiditis. Br J Ophthalmol. 2017;101(5):616–22.	17 eyes of 12 patients, (7 with PIC and 5 with MCP), out of which 9 patients longitudinally followed and 11 identified with CNV	Prospective, descriptive case series	OCTA identified CNV in cases where FA was inconclusive for the presence of CNV Correlation between OCT and OCTA findings as for suspected activity could confirm the diagnosis OCTA could be particularly helpful in distinguishing CNV from inflammatory lesions	Optovue Avanti-RTVue-XR (Optovue, Fremont, California, USA)
	Cheng L, Chen X, Weng S, Mao L, Gong Y, Yu S, Et Al. Spectral-domain optical coherence tomography angiography findings in multifocal choroiditis with active lesions. Am J Ophthalmol. 2016;169:145–61.	52 eyes of 26 MCP patients (14 bilaterally affected and 12 unilaterally affected)	Reliability and validity analysis (prospective)	20 of 23 active CNV cases were confirmed on OCTA 3 invalid OCTA results due to motion artifacts 32 of 34 inflammatory lesions in 13 eyes did not show blood flow in the outer retina on OCTA while the other 2 did OCTA could be helpful in distinguishing CNV from inflammatory lesions OCTA could not reliably give information about CNV activity (in contrast to FA) and thus, FA was still necessary to make therapy decisions in recurrent cases	Optovue RTVue XR Avanti (Optovue, Inc., Fremont, California, USA)
	Cerquaglia A, Lupidi M, Fiore T, Iaccheri B, Perri P, Cagini C. Deep inside multifocal choroiditis: an optical coherence tomography angiography approach. International Ophthalmology. 2017;37(4):1047–51.	1 eye of 1 patient	Single case report	Distinction of inflammation versus CNV is possible by multimodal imaging, especially OCTA was crucial for the therapeutic decision in this particular case OCTA may help in characterizing inflammatory lesions pattern and to detect the presence of CNV within a subretinal inflammatory or fibrotic tissue	OCTA prototype based on Spectralis OCT2 (Heidelberg Engineering, Heidelberg, Germany)
	Nakao S, Kaizu Y, Oshima Y, Sakamoto T, Ishibashi T, Sonoda Kh. Optical coherence tomography angiography for detecting choroidal neovascularization secondary to punctate inner choroidopathy. Ophthalmic Surg Lasers Imaging Retina. 2016;47(12):1157–61.	2 eyes of 1 patient	Single case report	OCTA detected abnormal flow in the outer retina, corresponding to type 2 CNV in PIC lesions OCTA showed remodeling of the choroidal capillaries and decreased flow in CNV after the treatment with anti-VEGF OCTA could be useful for the follow-up and evaluation of therapeutic strategies used to treat PIC and might prevent excessive administration of anti-VEGF therapy	Spectral-domain OCT (SD-OCT) (HRA; Heidelberg Engineering, Heidelberg, Germany)
	Astroz, P., Et Al., Optical coherence tomography angiography to distinguish choroidal neovascularization from macular inflammatory lesions in multifocal choroiditis. Retina, 2018. 38(2): P. 299–309.	18 eyes of 13 patients	Retrospective case series	Active CNV and inactive CNV were not significantly different concerning the different features on OCTA 5/15 (33.3%) eyes presented multifocal high-flow networks in the outer retina segmentation on OCTA images After anti-VEGF treatment, CNV size decreased on OCTA (but not statistically significant) OCTA can be useful in differentiating active inflammatory chorioretinal lesions from CNV, but not distinguish between active and inactive CNV	Optovue RTVue XR Avanti (Optovue, Inc., Fremont, California, USA)
MEWDS	Yannuzzi Na, Swaminathan Ss, Zheng F, Miller A, Gregori G, Davis Jl, Et Al. Swept-Source OCT angiography shows sparing of the choriocapillaris in multiple evanescent white dot syndrome. Ophthalmic Surg Lasers Imaging Retina. 2017;48(1):69–74.	2 eyes of 2 patients	Case reports	OCTA revealed choriocapillary circulation to be normal in the acute phase Suggestion that MEWDS is a result of an injury of the photoreceptors, and that hypofluorescence in ICGA imaging may be caused by irregular staining of tissue by extravasated ICG dye rather than a choroidal vascular abnormality	Swept-source OCTA, no details available
	Pichi F, Srvivastava Sk, Chexal S, Lembo A, Lima Lh, Neri P, Et Al. En face optical coherence tomography and optical coherence tomography angiography of multiple evanescent white dot syndrome: new insights into pathogenesis. Retina. 2016;36 Suppl 1:S178-S88.	29 eyes of 35 patients	Retrospective case series	Normal OCTA findings of the choroid and choroidal hypofluorescence in ICGA could be due to the reduced absorption capacity of malfunctioning RPE (theory of Chang et al.) Proposition that MEWDS is primarily the result of inflammation at the RPE and outer photoreceptor level leading to a “photoreceptoritis” Temporary loss of the inner and outer segments could be due to inflammation of the photoreceptor segments or the RPE with secondary photoreceptor involvement Hyperautofluorescent “spots” on FAF may be the result of either an unmasking effect of RPE autofluorescence or secondary to thickened RPE cells Hyperautofluorescent “dots” may represent disrupted and shed photoreceptor segments that stain with FA and/or associated Muller cell disruption	Optovue Avanti-RTVue-XR (Optovue, Fremont, California, USA)
	Pereira F, Lima Lh, De Azevedo Agb, Zett C, Farah Me, Belfort R, Jr. Swept-source OCT in patients with multiple evanescent white dot syndrome. J Ophthalmic Inflamm Infect. 2018;8(1):16.	2 eyes of 2 patients	Case reports	Normal OCTA findings of outer retina and of choriocapillaris during the acute phase of disease may suggest the primary injury in the outer retina and the photoreceptors	Spectral-domain-OCTA (DRI Swept Source OCT Triton, Topcon, Japan)
	Nozaki M, Hamada S, Kimura M, Yoshida M, Ogura Y. Value of OCT angiography in the diagnosis of choroidal neovascularization complicating multiple evanescence white dot syndrome. Ophthalmic Surg Lasers Imaging Retina. 2016;47(6):580–4.	1 eye of 1 patient	Case report	OCTA can be a useful tool and easier applicable than ICGA for diagnosing and follow-up of type 2 CNV in MEWDS, also in case of recurrence	Optovue Avanti-RTVue-XR (Optovue, Fremont, California, USA)
POHS	Liu Tya, Zhang Ay, Wenick A. Evolution of choroidal neovascularization due to presumed ocular histoplasmosis syndrome on multimodal imaging including optical coherence tomography angiography. Case Reports In Ophthalmological Medicine. 2018;2018:4098419.	2 eyes of 1 patient	Single case report	OCTA made CNV lesions visible when no CNV activity was seen on FA or SD-OCT at follow-up visits Suggestion that OCTA is more sensitive than FA or SD-OCT in detecting the presence of persistent CNV related to POHS The central trunk of CNV can be relatively resistant to anti-VEGF treatments Increase in CNV activity on OCTA does not necessarily lead to significant visual impairment	Spectral-domain-OCTA, no details available
APMPPE	Werner Ju, Enders C, Lang Gk, Lang Ge. Multi-modal imaging including optical coherence tomography angiography in patients with posterior multifocal placoid pigment epitheliopathy. Ophthalmic Surg Lasers Imaging Retina. 2017;48(9):727–33.	8 eyes of 4 patients	Case series	OCTA showed perfusion defects in choriocapillaris and choroid in APMPPE lesions OCTA perfusion deficits resolved in the choroid and later in the choriocapillaris over the course of the disease Suggestion that involvement of the choroidal circulation leads secondarily to damage of the outer retina OCTA might be able to replace FA and ICGA for diagnosing APMPPE	Cirrus 5000 equipped with the AngioPlex module (Carl Zeiss Meditec, Dublin, CA)
	Salvatore S, Steeples Lr, Ross Ah, Bailey C, Lee Rw, Carreno E. Multimodal imaging in acute posterior multifocal placoid pigment epitheliopathy demonstrating obstruction of the choriocapillaris. Ophthalmic Surg Lasers Imaging Retina. 2016;47(7):677–81.	2 eyes of 1 patient	Single case report	OCTA showed isolated choriocapillaris nonperfusion within APMPPE lesions in the active disease phase and improvement in flow over the course of the disease Choriocapillaris is suggested to be the primary focus of the disease	Optovue Avanti-RTVue-XR (Optovue, Fremont, California, USA)
	Maier M, Wehrmann K, Lohmann Cp, Feucht N. [OCT angiography findings in acute posterior multifocal placoid pigment epitheliopathy (Apmppe)]. Ophthalmologe. 2017;114(1):60–5.	2 eyes of 1 patient	Single case report	Hypofluorescent areas visible on ICGA in the early and late phases showed corresponding hypoperfused areas by OCTA of APMPPE lesions	Spectral-domain-OCTA, no details available
	Heifermann Mj, Rahmani S, Jampol Lm, Nesper Pl, Skondra D, Kim La, Et Al. Acute posterior multifocal placoid pigment epitheliopathy on optical coherence tomography angiography. Retina. 2017;37(11):2084–94.	10 eyes of 5 patients	Retrospective observational case series	In APMPPE lesions, conclusive resolvement of the choroidal vasculature on OCTA was difficult through overlying RPE thickening and inflammatory changes In 1 of 5 patients, choriocapillaris flow deficit extended outside the visible intraretinal lesion Recovery of choroidal flow parallel with healing of the APMPPE lesions Attenuated OCTA signal related to acute lesions was impossible to distinguish from primary choriocapillaris nonperfusion on OCTA Hypointensity on en face OCT could be due to projection artifacts and signal attenuation from chronic RPE thickening and pigmentary of acute and subacute lesions Suggestion of theory that isolated disruption to the choriocapillaris leaves the choroid in a sufficiently functional state to largely maintain the RPE/photoreceptor integrity Findings support decreased blood flow to be consistent with choriocapillary changes leading to a primary ischemic insult to the RPE rather than a primary RPE inflammatory etiology, but choriocapillaris changes could also be secondary to subclinical overlying RPE and retinal damage True course of RPE, retinal, and choroidal injury in APMPPE remains unclear	Optovue Avanti-RTVue-XR (Optovue, Fremont, California, USA) with split-spectrum amplitude-decorrelation angiography software
	Dolz-Marco R, Sarraf D, Giovinazzo V, Freund Kb. Optical coherence tomography angiography shows inner clumbrohoroidal ischemia in acute posterior multifocal placoid pigment epitheliopathy. Retin Cases Brief Rep. 2017;11 Suppl 1:S136-S43.	1 eye of 1 patient	Single case report	OCTA demonstrated inner choroidal flow improvement to accelerate after the initiation of oral prednisone Theory of primary choroidal pathophysiology is supported with OCTA findings Hypothesis, that a compensatory vascular mechanism is induced by the more central area of choroidal ischemia, because in the acute phase perilesional areas show increased inner choroidal flow that fades over time OCTA, compared to fundus examination, was more sensitive in detecting new lesions Compared to ultra-widefield dye-based angiographies, en face reconstructions of OCTA might be more sensitive, but conclusive findings outside the posterior pole are limited	Optovue Avanti-RTVue-XR (Optovue, Fremont, California, USA)
BSR	De Carlo Te, Bonini Filho Ma, Adhi M, Duker Js. Retinal and choroidal vasculature in birdshot chorioretinopathy analyzed using spectral domain optical coherence tomography angiography. Retina. 2015;35(11):2392–9.	8 eyes of 4 patients	Prospective observational review	On OCTA, BSR lesions showed areas of choriocapillaris flow reduction below retinal pigment epithelium disruption with larger choroidal vessels bordering or traversing those areas Suggestion of interpretation of those vessels being either a compensatory response by the choroid or being vessels from Sattler’s layer that are pushed into the choriocapillaris plane Flow reduction can be interpreted as greatly reduced blood flow or true choriocapillaris loss OCTA provided limited view of deeper choroidal layers OCTA showed abnormally tortuous vessels and increased intercapillary space in the retinal vasculature in all affected eyes 88% of affected eyes showed capillary loops and focal dilatations	AngioVue OCTA software on the com- mercially available Optovue Avanti-RTVue-XR (Optovue, Fremont, California, USA)
	Phasukkijwatana N, Iafe N, Sarraf D. Optical coherence tomography angiography of A29 birdshot chorioretinopathy complicated by retinal neovascularization. Retin Cases Brief Rep. 2017;11 Suppl 1:S68-S72.	2 eyes of 1 patient	Single case report	OCTA allowed quantification and specific distinction between superficial and deep capillary plexus pathology in BSR Diffuse reduction in the flow of both the SCP and DCP of the patient as compared with normal eyes, on OCTA OCTA demonstrated the DCP being more affected then the SCP Significant projection artifacts in the OCTA of the DCP were dealt with by adjusting and subtracting procedures DCP was considered to be more prone to hypoxic injury because if its blood supply	Optovue Avanti-RTVue-XR (Optovue, Fremont, California, USA)
	Pepple Kl, Chu Z, Weinstein J, Munk Mr., Van Gelder Rn, Wang Rk. Use of en face swept-source optical coherence tomography angiography in identifying choroidal flow voids in 3 patients with birdshot chorioretinopathy. Jama Ophthalmology. 2018;136(11):1288–92.	6 eyes of 3 patients	Prospective, longitudinal, observational case series	Choroidal flow voids in SS-OCTA ultra-widefield images colocalize with hypofluorescent ICGA lesions in BSR Suggestion that acute lesions are localized in Haller’s layer and chronic lesions may involve the entire choroid Hypothesis: true perfusion abnormality might be caused by choroidal vascular destruction in chronic disease, as opposite to flow voids in acute disease that might be due to change in the tissue’s optical properties by the presence of inflammatory cells	PLEX Elite 9000 (Carl Zeiss AG, Dublin, CA) (widefield images approximately 100°) were achieved by montaging sixteen 6 × 6-mm or five 12 × 12- mm cubes)
Serpiginous chorioiditis	El Ameen A, Herbort Cp, Jr. Serpiginous choroiditis imaged by optical coherence tomography angiography. Retin Cases Brief Rep. 2016;12(4):279–85.	2 eyes of 1 patient	Single case report	OCTA showed geographically shaped dark areas in lesions suggestive of decreased flow of the choriocapillaris OCTA could replace ICGA during some of the subsequent follow-up visits, whereas at initial evaluation, ICGA remains preferable Intermediate phase ICGA seemed to be more sensitive than OCTA for detecting hypoperfusion Hypoperfused areas diminished after cyclosporine was introduced	Optovue Avanti-RTVue-XR (Optovue, Fremont, California, USA)
	Montorio D, Giuffre C, Miserocchi E, Modorati G, Sacconi R, Mercuri S, Et Al. Swept-source optical coherence tomography angiography in serpiginous choroiditis. Br J Ophthalmol. 2017;102(7):991–95.	22 eyes of 11 patients	Retrospective cross-sectional and observational study	OCTA showed atrophy of choriocapillaris with an impairment of its detectable flow and greater visibility of choroidal vessels in inactive lesions OCTA showed no decorrelation signal in active lesions in choriocalipparis and the whole choroid Vessel density of the outer border of inactive lesions seemed to be lower than vessel density of unaffected areas	Swept-source OCTA (AngioPlex Elite 9000 SS-OCT, Carl Zeiss Meditech)
	Pakzad-Vaezi K, Khaksari K, Chu Z, Van Gelder Rn, Wang Rk, Pepple Kl. Swept-source OCT angiography of serpiginous choroiditis. Ophthalmology Retina. 2018;2(7):712–9.	6 eyes of 3 patients (4 eyes with choroidal lesions)	Prospective, observational case series	OCTA showed larger lesions of the choriocapillaris slab during active disease than in the outer nuclear layer slab and FAF areas Resolution of those lesions occurred, where OCTA findings were not associated with corresponding abnormal FAF (without clinical scarring after treatment) OCTA results seem to support the theory that the choriocapillaris is the primary site of pathology Suggestion of a simple grading system based on OCT, OCA and/or ICGA	Swept-source OCTA PLEX Elite 9000 (Carl Zeiss AG, Dublin, CA)
	Ahn Sj, Park Sh, Lee Br. Multimodal imaging including optical coherence tomography angiography in serpiginous choroiditis. Ocul Immunol Inflamm. 2017;25(2):287–91.	2 eyes of 1 patient	Single case report	OCTA demonstrated decreased vascularity on the choriocapillaris slab Loss of capillaries is partially replaced with irregular capillaris, but photoreceptor defect persisted following systemic corticosteroid therapy Choriocapillaris was seen as the main pathology of serpiginous choroiditis which may lead to the photoreceptor disruption	Swept-source OCTA, no details available
	Khan Ha, Shahzad Ma. Multimodal imaging of serpiginous choroiditis. Optometry And Vision Science: Official Publication Of The American Academy Of Optometry. 2017;94(2):265–9.	2 eyes of 1 patient (1 eye with choroidal lesions)	Single case report	OCTA revealed normal retinal architecture in lesions, but disruption of homogeneity of the choriocapillaris Role of choriocapillaris loss and hypoperfusion as a contributing factor towards the development of choroidal neovascularization in the later course is supported by OCTA findings Choriocapillaris was suggested to be affected most in earlier and later stages of the disease and its involvement might be the possible mechanism behind the development of CNVv in these cases	Swept-source OCTA, no details available
AMN	Aggarwal, K., A. Agarwal, D. Katoch, M. Sharma And V. Gupta (2017). “Optical coherence tomography angiography features of acute macular neuroretinopathy in dengue fever.” Indian J Ophthalmol 65(11):1235–1238.	2 eyes of 1 patient (1 eye with macular lesion)	Single case report	OCTA en face images showed disruption of both the SCP and DCP with flow deficit in the affected foveal region OCTA showed an increase of size of the FAZ SCP and DCP ischemia on OCTA did not change much at 6-month follow-up OCTA provided insights into the level of pathophysiological alterations in the affected eyes and helped in determining the visual prognosis	No details available
	Casalino, G., A. Arrigo, F. Romano, M. R. Munk, F. Bandello And M. B. Parodi (2019). “Acute macular neuroretinopathy: pathogenetic insights from optical coherence tomography angiography.” Br J Ophthalmol 103(3):410–414	11 eyes of 7 patients	Prospective, observational, cross-sectional study	OCTA revealed that global vascular perfusion is not impaired in the SCP and DCP, while a general flow void was found to be present in the choriocapillaris and focal impairment of the DCP Persistent difficulty to determine at which level the primary vascular insult occurs	PLEX Elite 9000 (Carl Zeiss AG, Dublin, CA)
	Kulikov, A. N., D. S. Maltsev And T. A. Leongardt (2018). Retinal microvasculature alteration in paracentral acute middle maculopathy and acute macular neuroretinopathy: a quantitative optical coherence tomography angiography study. Retin Cases Brief Rep. 10.1097/ICB.0000000000000709	6 eyes with PAMM and 2 eyes with AMN of 6 patients	Case series	OCTA revealed decreased vessel density of the SCP and DCP and changes in the shape of the superficial FAZ in PAMM and AMN eyes No difference was found in the FAZ area between PAMM/AMN eyes and healthy control eyes	Copernicus REVO (software Version 7.2; Optopol, Zawiercie, Poland)
	Lee, S. Y., J. L. Cheng, K. M. Gehrs, J. C. Folk, E. H. Sohn, S. R. Russell, Z. Guo, M. D. Abramoff And I. C. Han (2017). Choroidal features of acute macular neuroretinopathy via optical coherence tomography angiography and correlation with serial multimodal imaging. Jama Ophthalmol 135(11): 1177–1183.	9 eyes of 7 patients	Retrospective case series	OCTA revealed choriocapillaris flow void that colocalized to the AMN lesions in all affected eyes Using an automatic algorithm allowed a comparison between the areas of interest of two different imaging modalities using vascular registration OCTA did not reveal low abnormalities within the DCP Persistence of OCTA choroidal flow void after the resolution of the hyperreflectivity of the outer retinal layers Suggestion that decreased flow in choriocapillaris might be the primary insult in AMN, followed by hypoxic insult to the middle, then outer retina	AngioPlex, Cirrus HD-OCT 5000 (Carl-Zeiss Meditec Inc.)
	Nemiroff, J., D. Sarraf, J. P. Davila And D. Rodger (2018). Optical coherence tomography angiography of acute macular neuroretinopathy reveals deep capillary ischemia. Retin Cases Brief Rep 12 Suppl 1: S12-S15.	2 eyes of 1 patient (1 eye with macular lesion)	Single case report	OCTA showed flow deficit at the level of the DCP corresponding to the AMN lesion and OCT imaging	Optovue Avanti-RTVue-XR (Optovue, Fremont, California, USA)
	Thanos, A., L. J. Faia, Y. Yonekawa And S. Randhawa (2016). Optical coherence tomographic angiography in acute macular neuroretinopathy. Jama Ophthalmol 134(11): 1310–1314.	3 eyes of 2 patients	Case reports	OCTA did not reveal DCP nor SCP flow loss in AMN lesions Choriocapillaris flow abnormalities colocalised with the OCT alterations of the outer nuclear layer and ellipsoid zone identified with en face OCT, but were larger Suggestion that a vascular insult in the choriocapillaris is the pathogenic mechanism of the AMN lesions	OCTA device of Carl Zeiss Meditec, no details available
	Chu, S., P. L. Nesper, B. T. Soetikno, S. J. Bakri And A. A. Fawzi (2018). Projection-resolved OCT angiography of microvascular changes in paracentral acute middle maculopathy and acute macular neuroretinopathy. Invest Ophthalmol Vis Sci 59(7): 2913–2922.	22 eyes of 21 patients with 4 eyes being excluded (5 eyes with AMN and 13 eyes with PAMM)	Retrospective case series	On OCTA, PAMM lesions were associated with reduced MCP and DCP flow signals with partially additional reduced flow signal in the SCP PAMM eyes without later reperfusion of the MCP showed more severe inner nuclear layer thinning AMN lesions were associated with reduced DCP flow signals Conclusion that isolated focal DCP ischemia at the photoreceptor axons in the OPL is the trigger for AMN pathology, thereby leading to long-term outer nuclear layer thinning All AMN and PAMM lesions had evidence of variable recovery of capillary flow signal at the different capillary level	Optovue Avanti-RTVue-XR (Optovue, Fremont, California, USA)
	Pecen, P. E., A. G. Smith And J. P. Ehlers (2015). Optical coherence tomography angiography of acute macular neuroretinopathy and paracentral acute middle maculopathy. Jama Ophthalmol 133(12): 1478–1480.	2 eyes of 1 patient (1 eye with macular lesion)	Single case report	OCTA showed capillary nonperfusion correlating with subsequent inner nuclear layer atrophy in the presented case of PAMM OCTA could be used to differentiate laminar focal capillary defects that may be undetected by FA or when FA is not applicable for medical reasons in AMN and PAMM	Spectral-domain-OCTA, no details available
AZOOR	Levison, A. L., K. Baynes, C. Y. Lowder And S. K. Srivastava (2016). OCT angiography identification of choroidal neovascularization secondary to acute zonal occult outer retinopathy. Ophthalmic Surg Lasers Imaging Retina 47(1): 73–75.	1 eye of 1 patient	Single case report	Distinctive changes identified on OCTA aided in the diagnosis and management of CNV in AZOOR Suggestion that OCTA can be useful in distinguishing inflammatory changes versus neovascular changes in patients with posterior uveitis	Optovue Avanti-RTVue-XR (Optovue, Fremont, California, USA)
	Mehrotra, N., M. Nagpal, J. Khandelwal And R. Juneja (2018). Panoramic Optical Coherence Tomography Angiography Features In Acute Zonal Occult Outer Retinopathy. Indian J Ophthalmol 66(12): 1856–1858.	2 eyes of 1 patient	Single case report	Panoramic OCTA showed increased decorrelation signal at the DCP in the watershed zone of AZOOR lesions	RS-3000 Advance OCT (Nidek, Japan)
	Naik, A. U., N. Ezhilvathani And J. Biswas (2018). Acute zonal occult outer retinopathy: is optical coherence tomography angiography useful? Indian J Ophthalmol 66(11): 1637–1639.	2 eyes of 1 patient	Single case report	En-face OCTA images demonstrated hyperreflective dot structures at at the level of ellipsoid zone at presentation Vasculature of the SCP, DCP, choriocapillaris, and the choroid at presentation and over two consecutive monthly follow-ups was within normal limits Rise of question, whether AZOOR was part of pachychoroid spectrum Speculation that the en-face OCTA findings might represent the degenerating photoreceptor segments	AngioPlex (Carl Zeiss Meditec Inc., Dublin, CA, USA)
Miscellaneous	Wang Jc, Lains I, Sobrin L, Miller Jb. Distinguishing White Dot Syndromes Aith Patterns of Choroidal Hypoperfusion on Optical Coherence Tomography Angiography. Ophthalmic Surg Lasers Imaging Retina. 2017;48(8):638–46.	7 eyes of 4 patients with different WDS (APMPPE, BCR, MEWDS, POHS)	Case reports	APMPPE: hypoperfusion was more pronounced in the choriocapillaris than in the deeper choroid in OCTA and more widespread than the retinal lesions BCR: abnormalities in perfusion, that were more pronounced in the choroid than in the choriocapillaris; smaller areas of choroidal hypoperfusion did not have corresponding fundus lesions making OCTA probably a more sensitive diagnostic tool for early lesions MEWDS: well-demarcated focal areas of hypoperfusion in the choriocapillaris of larger lesions corresponded well to the white dots, which supports the hypothesis that the outer retina is the location of primary pathology POHS: focal injury appeared to occur in POHS as opposed to more widespread immune response in BCR, APMPPE, and MEWDS. OCTA revealed the correlation between the “punched out” lesions of POHS and hypoperfusion of choriocapillaris and choroid OCTA revealed hypoperfusion of the choriocapillaris and choroid as a shared feature among different white dot syndromes and the differences in the distribution, depth, and extend of these OCTA changes may aid in diagnosis	AngioPlex OCT Angiography (Carl Zeiss AG, Oberkochen, Germany) or Optovue Avanti-RTVue-XR (Optovue, Fremont, California, USA)
	Mangeon M, Zett C, Amaral C, Novais E, Muccioli C, Andrade G, Et Al. Multimodal evaluation of patients with acute posterior multifocal placoid pigment epitheliopathy and serpiginous choroiditis. Ocular Immunology And Inflammation. 2018;26(8):1212–18.	8 eyes of 4 patients (6 eyes with serpiginous choroiditis and two eyes with acute posterior multifocal placoid pigment epitheliopathy)	Case reports	APMPPE: OCTA showed, despite of the stage of disease, choriocapillaris hypoperfusion, but partial reperfusion in the choriocapillaris 2 months after treatment in one case Serpiginous choroiditis: OCTA allowed the diagnosis of CNV; RPE atrophy (as clarified on OCT and FAF) corresponded to localized OCTA hypoperfusion pattern of the choriocapillaris, which could generate a quantitative parameter usable for follow-up	Optovue Avanti-RTVue-XR (Optovue, Fremont, California, USA)

*AMN* acute macular neuroretinopathy, *anti-VEGF* anti-vascular endothelial growth factor, *APMPPE* acute posterior multifocal placoid pigmentepitheliopathy, *AZOOR* acute zonal occult outer retinopathy, *BSR* birdshot chorioretinopathy, *CNV* choroidal neovascularization, *DCP* deep capillary plexus, *FA* fluorescein angiography, *FAF* fundus autofluorescence, ICG indocyanine green, *ICGA* indocyanine green angiography, *MEWDS* multiple evanescent white dot syndrome, *MCP* multifocal choroiditis and panuveitis, *OCT* optical coherence tomography, *OCTA* optical coherence tomography angiography, *PAMM* paracentral acute middle maculopathy, *PIC* punctate inner choroidopathy, *POHS* presumed ocular histoplasmosis syndrome, *RPE* retinal pigment epithelium, *SCP* superficial capillary plexus, SD-OCTA spectral domain OCTA, *SS-OCTA* swept-source OCTA

#### Acute posterior multifocal placoid pigment epitheliopathy (APMPPE)

**Fig. 3:**
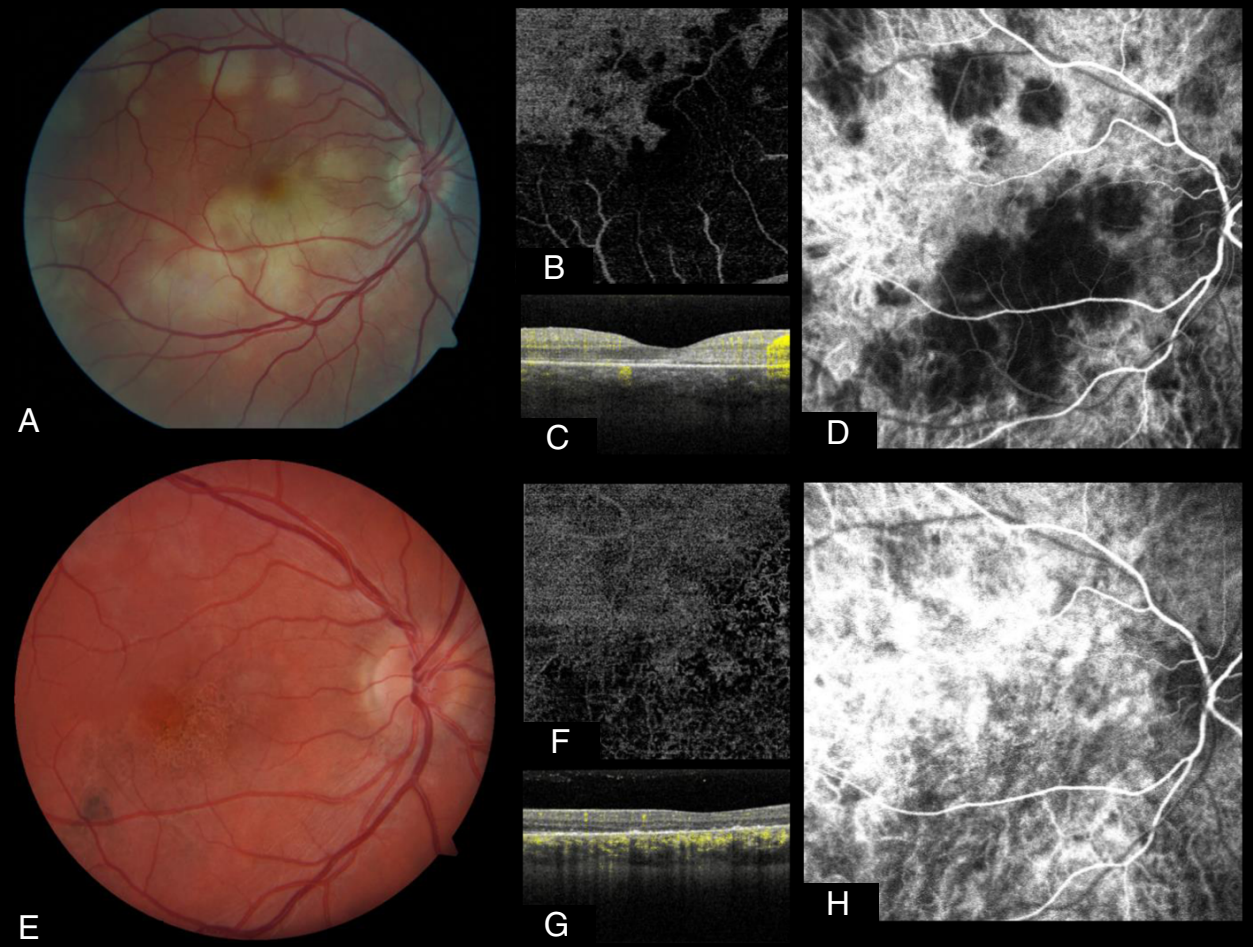
Multimodal imaging analysis of the macular and peripapillary area of the right eye showing multiple lesions at different stages of acute posterior multifocal placoid pigment epitheliopathy (APMPPE). Images **a**, **b**, **c** and **d** were taken 3 days after onset of symptoms. **a** Color image of the fundus showing fresh lesions. **b** En face reconstruction of the choriocapillaris flow by optical coherence tomography angiography (OCTA) demonstrating an area of lacking signal. **c** Structural OCT with superimposed flow signal revealing areas with decreased flow signal. **d** Early phase indocyanine green angiography (IA) with a hypocyanescent area corresponding to clinically visible early lesions. Images **e**, **f**, **g**, and **h** were taken 6 months after the onset of symptoms. **e** Color image of the fundus showing old macular lesions. **f**, **g** En face reconstruction of the choriocapillaris flow by OCTA demonstrating an increased area of low signal compared to **b** and **c**, respectively. **g** Structural OCT with superimposed flow signal revealing an increase of low signal compared to **c**. **h** Early-phase IA showing almost no sign of the hypocyanescent area seen in **d**

APMPPE or multifocal ischemic choroidopathy is a rare type of uveitis, which is characterized by light yellow lesions localized in the outer retina, the retinal pigment epithelium (RPE), and the choroid, due to inflamed choriocapillaris, RPE, and outer layers of the retina. Typical FA findings in the acute phase of the disease are an early blockage of the choroidal background fluorescence under the active lesions, and late staining, whereas in ICGA, these lesions are hypocyanescent during early and late phases of the disease, interpretable as hypoperfusion of the choriocapillaris. In the later course of the disease, late phase FA exhibits mottling hyperfluorescence, staining, and window defects due to scarring of RPE, whereas ICGA patterns remain the same. Early in the course of the disease, OCTA shows normal perfusion patterns of the superficial and deep layers of the retina, but reduced perfusion of the choriocapillaris and choroid like, not unexpectedly, in FA and ICGA, and also as areas of altered FAF [59–61]. Over time, the extent of the non-perfused areas decreases and signs of vascular reperfusion can be noted, but persistent flow void remains at the level of the choriocapillaries [62, 63]. A multimodal imaging example comparing early and late stage of the disease is given in Fig. 3. Disease-specific limitations of the method are its limited ability to give flow information of the peripheral retina and the limited resolution of the choroidal vasculature, due to the overlying thickened RPE and further inflammatory changes.

#### Serpiginous choroiditis

Serpiginous choroiditis is a rare ocular pathology that presents with binocular, but asymmetric grayish or yellow inflammatory lesions, which develop in an irregular serpentine pattern. These lesions develop into an atrophy of the outer retina, RPE, and the choriocapillaris.

In FA, acute lesions show early hypofluorescence and late leakage and staining at lesions’ border. Older lesions present with window defect, and, in case of progress or relapse, with staining along the progressing edges. ICGA has been used for staging and detecting new activity during the course of the disease. The area of hypocyanescent choroidal non-perfusion is usually larger than expected by fundus examination, and OCTA can be used in serpiginous choroiditis to detect subclinical or persistent choroidal nonperfusion as lesion size on the OCTA choriocapillaris slabs shows a good correlation with lesions on ICGA [64]. In active disease, the flow void area of the choriocapillaris slabs not associated with corresponding abnormal autofluorescence on FAF resolve without sequela [65].

As reported in another retrospective study with 22 eyes and case series of six eyes, there was no flow signal throughout the entire choroid in active lesions, whereas in inactive lesions, OCTA signals indicated some hyperintensity, which is interpreted as rarefaction of choroidal vessels and loss of choriocapillaris [60, 66].

#### Multiple evanescent white dot syndrome (MEWDS)

OCTA also helped to get a better understanding of the pathogenesis of MEWDS. In contrast to serpiginous choroiditis and APMPPE, OCTA revealed that the choriocapillaris is not involved in MEWDS. MEWDS is characterized by gray-white fundus lesions, which are the result of RPE lesions, typically located outside the fovea in a granular pattern. Rare findings can be sheathing of retinal veins and superficial retinal hemorrhages.

In MEWDS lesions, an early wreath-like hyperfluorescence of the smaller “dots” and late staining of larger “spots” on FA can be seen.

On FAF images, hyperautofluorescent dots can be appreciated even when funduscopy does not correlate with these dots. On ICGA, the early phase is unremarkable, but in the late phase, multifocal hypocyanescent lesions, even in clinically inconspicuous areas, with ill-defined margins, characterize the lesions of MEWDS. Looking at the unremarkable choriocapillaris of OCTA findings in MEWDS, hypocyanescence in ICGA could no longer be interpreted as changes of choroidal vascularity, but supported the thesis of Chang et al., mentioned above, that under physiological conditions, RPE normally absorbs ICG, causing physiological background hypercyanescence, and thus, it has been hypothesized that in MEWDS, the RPE is primarily affected and that ICGA uptake is interrupted by diseased RPE [21, 25, 67–69]. However, in larger lesions of MEWDS, reduced flow of choriocapillaris had also been detected, thus ICGA hypocyanescence may be still interpreted as a reduction of choroidal blood flow [70]. However, altered flow information may be also due to a change in the optical property of the choroid due to blockage of invading inflammatory cells. Once again, the limitation of OCTA in detecting flow in the slow-flow system of the choriocapillaris with high enough sensitivity needs to be taken into account [71].

Remarkably, in the light of OCTA findings in this uveitis type, the discussion about the pathogenesis of MEWDS was recently risen up and mainly questions the primary focus of inflammation, be it in the RPE or the choriocapillaris [70].

#### Multifocal choroiditis and panuveitis (MCP)

MCP, a chronic, recurrent disease, typically affects female patients in their fourth decade of life. Multiple yellow or yellowish gray lesions of various size, primarily at the level of the RPE and inner choroid, in association with vitreous cells, can be seen on funduscopy.

**Fig. 4:**
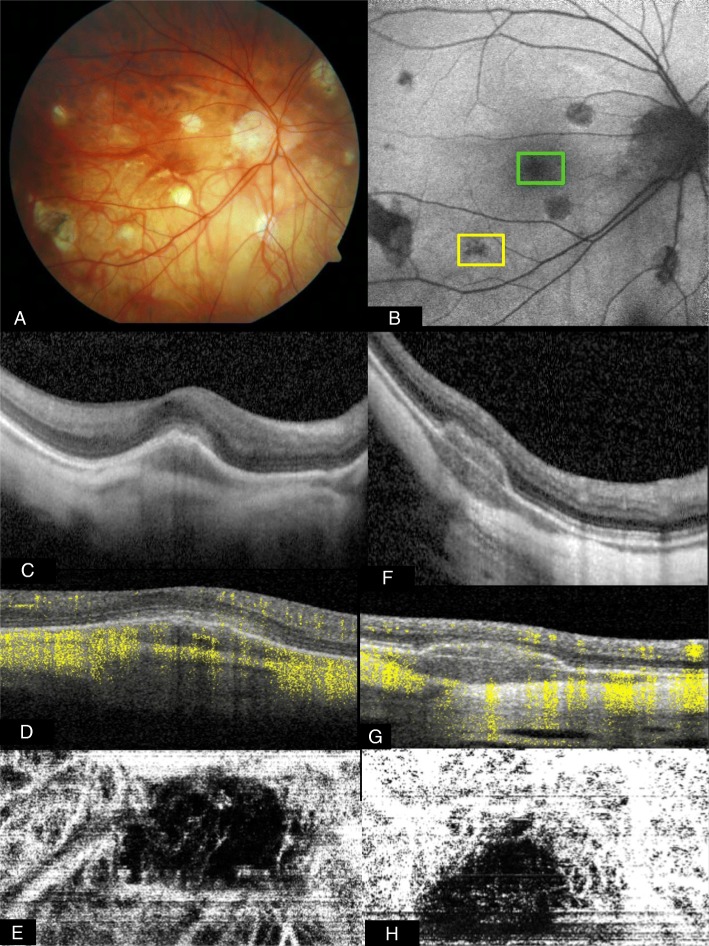
Optical coherence tomography angiography (OCTA) for differentiating acute inflammatory lesions from choroidal neovascularization in a myopic eye with idiopathic multifocal choroiditis. **a** Color photograph shows multiple yellowish-gray lesions perifoveolar and nasal the optic nerve head. **b** Fundus autofluorescence in this case shows unspecific mottled hypoautofluorescence of the lesions. **c** Structural OCT scan over the lesion within the green box in panel B shows hyperreflective material below and minimal above the retinal pigment epithelium (RPE). **d** Cross-sectional OCTA of the same lesion demonstrates slightly abnormal flow above the RPE, representing type 2 neovascularization. **e** En face OCTA reconstruction, which shows a small choroidal neovascularization. **f** Structural OCT scan over the lesion within the yellow box in panel **b** shows hyperreflective material below the RPE and a well-defined Bruch’s membrane. **g** Cross-sectional OCTA shows the absence of abnormal flow beneath the RPE. The flow overlying the RPE layer represents the projection of the flow signal from the more superficial retinal circulation (projection artifact). **h** En face OCTA reconstruction. It looks like vascular branches of a choroidal neovascularization, but scrolling the slap through the retina represents a projection artifact

In their inactive form, these lesions appear punched out, possibly with pigmented fraction and scarring. A common complication in MCP is an inflammatory CNV of type 2 and, as already noted above, OCTA can be very helpful in these cases to define and detect inflammatory CNVs, which is essential for further treatment decisions [15, 19, 54, 55, 57, 72] (Fig. 4).

But OCTA has also been shown to have limitations in differentiating active neovascularization versus an inactive scar [57]. Again, the result of an OCTA examination cannot be interpreted without considering conventional OCT and dye-based imaging techniques.

#### Punctate inner choroidopathy (PIC)

Similarly to MCP, PIC is a condition in which early detection of new and progressing inflammatory CNV is of high relevance, as the development of macular or foveal CNV in PIC is a common complication, even more common than in MCP. In contrast to MCP, PIC rarely is associated with vitritis and lesions are less commonly seen anterior to the posterior pole and are rather scattered than arranged in a particular pattern. Similarly to MCP, it has been reported that OCTA can detect inflammatory CNV, when FA may be not conclusive due to a similar appearance of a type 2 CNV and an active lesion [15, 19, 54, 57, 73].

#### Presumed ocular histoplasmosis syndrome (POHS)

OCTA reports on POHS are very rare [70, 74]. POHS is characterized by multiple white atrophic chorioretinal scars or spots, so-called “histo-spots,” peripapillary atrophy and the absence of vitritis. It may be associated with CNV. Sixty percent of the cases are bilateral. POHS lesions correspond to areas of focal flow loss in the choriocapillaris and deeper choroidal layers on OCTA, which is consistent with the theory that choroidal seeding of *Histoplasma capsulatum* spores leads to the destruction of choroidal blood vessels [70].

In contrast to most other white dot syndromes, the lesions are more focal than widespread. Only one case of CNV due to POHS based on OCTA evolution has been published until today, and it has been suggested that, in POHS, OCTA might be more sensitive than FA or OCT in detecting very early increase or recurrence of CNV activity [74].

#### Birdshot retinochoroidopathy (BSR)

The name BSR was given to this form of white dot lesion due to its shotgun pattern of creamy whitish lesions scattered throughout the fundus. It is strongly associated with HLA-29 and presents with moderate vitritis and phlebitis, and the lesions affect the choroid and the retina. In a prospective observational review of four patients with BSR, OCTA visualized telangiectasia, capillary dilatations and loops, and an increased intercapillary space in the retinal vasculature [75]. Moreover, looking at the SCP in birdshot patients, OCTA showed a larger area of FAZ than previously supposed using FA [19]. Furthermore, as central retinal thinning in birdshot patients correlates with these areas displaying a decreased capillary perfusion on OCTA, it was postulated that thinning of the macula could be due to an ischemic insult to ganglion cells and their axons, although macular thinning on OCT scans show mainly a thinning of outer retinal layers [19]. Looking at the DCP, even more reduction of flow could be detected, which was proposed to be, besides inflammatory reasons, a possible explanation for the development of retinal neovascularization in BSR [76] (Fig. 1).

OCTA demonstrates multifocal hypoperfusion within the choriocapillaris and even larger areas of hypoperfusion when looking at deeper choroidal layers [70, 75]. Early active lesions are found in near vicinity of the larger vessels of the Haller layer [68]. While early active lesions may only involve the deeper choroidal layers and may regress under treatment, chronic birdshot lesions will reveal full thickness choroidal flow void also affecting the choriocapillaris. These lesions will not regress under appropriate treatment [77]. Based on these findings, OCTA may be a reliable additional tool to follow birdshot patients and to identify disease progression and insufficient treatment [77].

#### Acute macular neuroretinopathy (AMN)

AMN is characterized as wedge-shaped superficial retinal defects in the macula, resulting in paracentral scotomas and are associated with vasoconstrictors and sympathomimetics. The fact that a majority of patients are young females and a lot report flu-like symptoms prior onset of eye symptoms has prompted the conclusion that AMN belongs to the spectrum of white dot syndromes. On OCT, AMN starts with hyperreflectivity of the outer plexiform layer with subsequent thinning of the outer nuclear layer and a significant attenuation of the interdigitation zone (IZ) and ellipsoid zone and external limiting membrane. Changes resolve over time; however, usually, sequelae of IZ attenuation, photoreceptor layer thinning, and corresponding scotomas remain. In various case reports or series, OCTA examination revealed a flow void in the DCP [78, 79]. However, some case series also found persistent flow attenuation in the choriocapillaris [78, 80, 81]. While paracentral acute middle maculopathy (PAMM) shows consistently flow changes in the DCP as well as the middle capillary plexus and occasionally also in the SCP throughout the literature, the changes found in AMN are more inconsistent [82–85]. Based on these findings, it is hypothesized that the location of primary insult in AMN is located in the DCP [84]. In contrast to AMN, PAMM is often associated with cardiovascular diseases and cardiovascular risk factors and should therefore not be restricted to the white dot spectrum [81, 86].

#### Acute zonal occult outer retinopathy (AZOOR)

AZOOR is a rare retinopathy that, in some cases with preceding viral infection, affects one eye, but may also progress to involvement of the second eye. Intraocular cells are rare and the fundus in the early phase appears normal. As the disease progresses, decreased vision and photopsia is realized by the patient, and a scotoma coinciding with the affected zone of outer retinal dysfunction, often an enlargement of the blind spot, is detectable. The typical finding in progressing AZOOR lesions is retinal atrophy and mottling of the RPE leading to typical fundus changes with grayish or yellowish lines along the atrophy area of the RPE, outer retina, and choroid. FAF illustrates a characteristic trizonal sign with the increased autofluorescence of active lesions and decreased autofluorescence of damaged RPE cells and normal autofluorescence of the unaffected retina. ICGA may be normal or show hypocyanescence. FA shows, later in the course of the disease, window defects. OCTA findings may show increased decorrelation signal at the DCP at the watershed zone [87], but at early presentation, a case report demonstrated normal OCTA imaging of the retinal and choroidal vasculature, whereas en face OCTA structural analysis revealed hyper-reflective dots at the level of ellipsoid zone of different patterns in both affected eyes of one patient [88].

### About optical coherence tomography angiography in infectious uveitis

Most existing OCTA reports on infectious uveitis types are related to tuberculosis (TB)-associated uveitis. Furthermore, there are some reports on toxoplasmosis, acute retinal necrosis (ARN), and more infrequent conditions such as dengue fever, Bartonella (cat-scratch disease) and West Nile virus infection, which will be summarized below.

#### Intraocular tuberculosis (TB)

TB has various forms of appearance: granulomatous anterior uveitis, intermediate uveitis, panuveitis or posterior uveitis, the latter being the most common presentation. Diverse clinical findings can be seen in TB, e.g., granulomata, occlusive retinal vasculitis, or serpiginous-like choroiditis or multifocal serpiginoid choroiditis [89]. In TB, OCTA has proven to be useful in the detection of CNV in a patient with tubercular serpiginous-like choroiditis and showed an involvement of various retinochoroidal layers by an abnormal vascular network [90].

In serpiginous-like TB chorioretinitis, OCTA showed a more precise picture of choroidal hypoperfusion as compared with ICGA [89] and was useful in the follow-up of patients with tubercular multifocal serpiginoid choroiditis [91].

In a case report of choroidal granuloma caused by TB, a type 2 CNV could be detected in its early stage, and growth was documented with the help of OCTA, although secondary complications like intraretinal fluid were not yet found [52].

In a sample of nine eyes with different forms of posterior tuberculous uveitis, OCTA showed neovascular flow assigned to type 1 CNV [53].

For longitudinal choriocapillaris flow evaluation in a TB patient with serpiginous-like choroiditis, OCTA showed progressive restoration of the choroidal circulation over time, with reduction of the lesion size and reduction in hypoperfusion, suggesting vascular remodeling during the recovery phase [92]. The choriocapillaris flow appeared to progressively restore centrally from the lesion edges under treatment, correlating with FAF signal changes over time, which is an interesting and similar finding as in APMPPE and in idiopathic serpiginous chorioretinopathy [92]. These findings were confirmed in a larger prospective case series of 32 eyes using a panoramic OCTA device [93].

#### Toxoplasmosis

The parasite *Toxoplasma gondii*, an intracellular protozoan, causes necrotizing retinochoroiditis and can cause severe vision loss. A possible complication is CNV, which can be detected quite easily with OCTA (Fig. 2). A double case report about CNV in toxoplasmosis confirmed the utility of OCTA for CNV secondary to ocular toxoplasmosis, especially, as FA can be difficult to interpret in a scarred area [94].

Another, less recent case report focused on the advantage of volume rendering on OCTA of such lesions, which allows visualization of all OCTA scans simultaneously [95].

A new case report used OCTA for longitudinal follow-up of a toxoplasmic lesion and showed vascular obliteration of the retina and choroid in the acute stage with only partial restoration in the periphery of the lesion during the quiescent stage, but with persistence of destruction in the choriocapillaris texture [96].

#### Herpesviridae

Acute retinal necrosis (ARN), mainly caused by varicella zoster virus and herpes simplex virus types 1 and 2, is characterized by vitreal and anterior chamber inflammatory reaction, retinal necrotic lesions, circumferential progression, and evidence of occlusive vasculopathy. A recent case report suggested that OCTA could be used in ARN cases to evaluate the therapeutic response and anatomical improvement as macular vascular density has been observed to decrease until normal density is restored within one month in the SCP and DCP during therapy, supplied by capillary branches of the vessel embedded in a necrotizing retinal lesion [97].

#### Dengue fever

Dengue fever, an arthropod-borne disease, caused by four serotypes of Dengue virus, can manifest various ophthalmologic symptoms, such as subconjunctival hemorrhage, keratitis, anterior uveitis, angle-closure glaucoma as for anterior segment and, more commonly, ME, retinal hemorrhages, foveolitis, cotton wool spots, AMN lesions, and microaneurysms as for posterior segment involvement [98]. Deduced from case reports, OCTA has proven to be a worthy diagnostic tool for detecting flow deficits in the perifoveal region within the DCP and SCP in ischemic inflammatory foveolitis and outer maculopathy, even when fundus lesions were not detectable [98–100].

#### Cat-scratch disease

Bartonella henselae causes focal retinochoroiditis, Parinaud oculoglandular syndrome and neuroretinitis. Bartonella bacteria tend to colonize endothelial cells which seem to have vasoproliferative effects and local CNV-development [101]. The case of one patient with Bartonella-associated CNV was investigated with OCTA, which proved to be helpful, since dye-based angiographies could not be interpreted conclusively as the hyperfluorescence of the inflammatory lesion did not allow the differentiation of a local neovascular complex [101].

#### West Nile virus (WNV)

West Nile virus (WNV) infection of the eye is mostly associated with bilateral multifocal chorioretinitis. Vascular involvement has also been described, including retinal hemorrhages, retinal vascular sheathing, retinal vascular leakage on FA, and, rarely, mostly in patients of advanced age with diabetes, occlusive retinal vasculitis. Of the latter form, one case report, already mentioned above, has been published, and the authors claimed the superiority of OCTA to FA for detecting ischemic retinal capillary changes, allowing a differentiation between involvement of the SCP and the DCP, with the latter one being more involved, as also described earlier in the case of BSR and Behçet’s uveitis [45–47]. While during follow-up, funduscopic lesions diminished, retinal capillary nonperfusion on OCTA persisted [45].

## Conclusion

In recent years, many articles about the application of OCTA in eyes with various forms of uveitis have been published, with a major part of them being case reports or case series. Obviously, the problem is twofold. First, specific algorithms to interpret specific issues need to be developed to make its use more attractive for clinicians. Second, OCTA has not yet been established in the daily routine of ophthalmologists, suggesting that the development and implementation of such algorithms will be slow, as is to be expected with a newly introduced diagnostic tool. The latter reason might also be due to the fact that knowledge and understanding of artifacts is required in order to enable the clinician to make valid interpretations of the OCTA images, even though the handling and transposition per se could be well suited for daily use.

The use of OCTA in inflammatory CNV, for example, seems to be one of the easier parts for interpretation, which is natural given inflammatory CNV being a lesion of many uveitis types and also the widespread use of OCTA in AMD related CNV. However, the number of various uveitis cases will not easily allow larger sample size studies.

A good example of this problem is white dot syndromes. Here, studying their different subtypes, specifically with OCTA, is challenging because of their fairly rare prevalence. However, OCTA might be of great relevance, as their classification system and, in some cases also the theory about their pathogenesis, might have to be re-evaluated.

In summary, OCTA can be seen as a complementary diagnostic tool in uveitis, which, in some cases, can highlight pathology earlier in the course of the disease than traditional imaging tools and sometimes gives an indication of the affected layers in or under the retina, which were formerly not detectable. Of course, the awareness of typical artifacts should always be kept in mind during the interpretation of the images, and future studies with larger sample sizes should aim at elaborating on further disease-specific algorithms.

## Abbreviations

AMN: Acute macular neuroretinopathy
anti-VEGF: Anti-vascular endothelial growth factor
APMPPE: Acute posterior multifocal placoid pigmentepitheliopathy
ARN: Acute retinal necrosis
AZOOR: Acute zonal occult outer retinopathy
BSR: Birdshot chorioretinopathy
CNV: Choroidal neovascularization
DCP: Deep capillary plexus
DSF: Diffuse subretinal fibrosis
EDI-OCT: Enhanced depth imaging-OCT
FA: Fluorescein angiography
FAF: Fundus autofluorescence
ICG: Indocyanine green
ICGA: Indocyanine green angiography
IU: Intermediate uveitis
IZ: Interdigitation zone
MCP: Multifocal choroiditis and panuveitis
ME: Macular edema
MEWDS: Multiple evanescent white dot syndrome
OCT: Optical coherence tomography
OCTA: Optical coherence tomography angiography
PAMM: Paracentral acute middle maculopathy
PIC: Punctate inner choroidopathy
POHS: Presumed ocular histoplasmosis syndrome
RPE: Retinal pigment epithelium
SCP: Superficial capillary plexus
SD-OCTA: Spectral domain OCTA
SS-OCTA: Swept-source OCTA
SSPiM: Suspended scattering particles in motion
TB: Tuberculosis
VKH: Vogt-Koyanagi-Harada disease
WNV: West Nile Virus
